# Effects of exercise training on vasomotor symptoms and quality of life in postmenopausal women: a randomized controlled trial

**DOI:** 10.1186/s12905-025-04231-y

**Published:** 2025-12-22

**Authors:** Gizem Yilmaz Babacan, Sebile Guler Cekic, Zeliha Candan Algun, Ahmet Fatih Durmusoglu

**Affiliations:** 1 Graduate School of Health Sciences, Physiotherapy and Rehabilitation, Istanbul Medipol University, Istanbul, Turkey; 2 Department of Obstetrics and Gynecology, Koc University Hospital, Istanbul, Turkey; 3 International Faculty of Medicine, Department of Obstetrics and Gynecology, Istanbul Medipol University, Istanbul, Turkey

**Keywords:** Resistance exercise, Aerobic exercise, Menopausal symptoms, Rehabilitation, Hot flushes

## Abstract

**Background:**

The impact of structured exercise interventions on vasomotor symptoms (VMS), quality of life, and sleep disturbances in postmenopausal women is inconclusive. This study aimed to evaluate the effects of a 12-week program combining resistance and aerobic training on these outcomes.

**Methods:**

Thirty-eight postmenopausal women aged 45–65 years with moderate or severe VMS were randomized to a 12-week combined resistance and aerobic training program (*n* = 20) or a control group (*n* = 18). Resistance exercises using body weight were performed twice weekly, and moderate-intensity outdoor walking three times weekly. The primary outcome was the change in VMS severity from baseline to week 12, assessed by the vasomotor domain of the Menopause-Specific Quality of Life (MENQOL) questionnaire. Secondary outcomes were changes in quality of life and sleep quality assessed with the MENQOL, Menopause Rating Scale (MRS), and Pittsburgh Sleep Quality Index (PSQI). Data were analyzed using mixed-model repeated-measures ANOVA to compare within- and between-group changes.

**Results:**

Of the 38 participants enrolled, 36 completed the trial. The exercise group showed a large improvement in VMS severity (MENQOL vasomotor domain, η²=0.81, *p* < 0.001) and in somatic symptoms (MRS somatic domain, η²=0.62, *p* < 0.01). Significant improvements were also observed in the MENQOL psychosocial and physical domains, MRS psychological symptoms, and PSQI sleep quality (all *p* < 0.01). No significant between-group differences were found in the sexual domains of either scale (*p* > 0.05).

**Conclusions:**

A combination of resistance and aerobic exercise training is an effective way to alleviate menopausal symptoms, particularly the severity of VMS, and improve mood and sleep quality in postmenopausal women.

**Registry:**

ClinicalTrials.gov, *Trial registration*: NCT05892094, *Registration date*: 17 May 2023.

**Supplementary Information:**

The online version contains supplementary material available at 10.1186/s12905-025-04231-y.

## Background

Vasomotor symptoms (VMS), which are characterized by hot flushes and night sweats, are the most reported symptoms of menopause transition, affecting 85% of women at some point during this period. VMS are experienced as hot flushes, excessive sweating, flushing, and a feeling of intense heat in the face, head, and neck regions, and seriously affect women’s quality of life [1]. VMS are associated with adverse health outcomes such as poor cardiovascular health, disrupted sleep, and increased fatigue, and can negatively impact work performance, daily activities, and social relationships [2, 3].

Although menopausal hormone therapy (MHT) remains the most effective pharmacological treatment for VMS, it may not be suitable or preferred by all women due to contraindications or side effects. Consequently, safe, evidence-based, non-pharmacological approaches are increasingly explored as complementary or alternative options [4, 5].

Exercise has proven to be effective for improving quality of life in middle-aged women in an easy and non-invasive way, but the evidence remains inconsistent [6, 7]. A structured and planned exercise program is seen as a potential alternative treatment for VMS. Emerging evidence shows that structured exercise, including aerobic and resistance interventions, has both positive and limited effects on reducing menopausal symptoms [5, 8]. However, findings across studies remain mixed, with some trials reporting a reduction in symptoms and others showing no improvement or even transient worsening during or after activity [9].

The hormonal changes that occur during in menopause cause thermoregulatory instability [10]. As estrogen levels decrease, hypothalamic KNDy (kisspeptin, neurokinin B, and dynorphin) neurons become overactivated, leading to increased neurokinin B and NK-3 receptor signalling, which stimulates the autonomic thermoregulatory pathways responsible for vasodilation and heat dissipation [11] Exercise may help restore thermoregulatory balance by modulating endorphin release and central nervous system activity, potentially explaining the observed reduction in the intensity of hot flushes with regular physical activity [12].

Previous studies investigating the effects of exercise on VMS have reported inconsistent results. While some trials have shown reductions in hot flush severity and improved quality of life, others have demonstrated no significant effect, or even a temporary worsening of symptoms. For instance, Daley et al. [12] and Newton et al. [13] observed limited or no improvement following moderate-intensity aerobic programmes, whereas Elavsky and McAuley [14] reported benefits primarily associated with mood and sleep rather than direct symptom relief. These inconsistencies may be due to differences in the type of exercise, level of supervision, and characteristics of the participants, as most previous trials focused solely on aerobic training or self-directed physical activity. There is limited evidence regarding the impact of structured, supervised programmes combining resistance and aerobic exercises, particularly among women experiencing moderate to severe VMS [8]. Addressing this gap is essential in order to better define safe, feasible, and effective non-pharmacological strategies for managing menopausal symptoms.

The aim of this study was to investigate the effects of resistance exercises combined with aerobic exercises on VMS, quality of life, and sleep disturbance. We hypothesized that 12 weeks of exercise training would decrease moderate and severe VMS more than in a control group receiving no intervention.

## Methods

### Study design

This open, assessor-blinded, parallel-group, randomized controlled trial aimed to assess the effect of exercise training on the frequency of VMS in postmenopausal women. Participants were allocated at a ratio of 1:1 to either a 12-week combined resistance and aerobic exercise training program or a control group. The assessor who administered and scored the questionnaires was blinded to group allocation and did not participate in the intervention process. Data were coded prior to analysis to maintain evaluator blinding. Clinical Trial approval was obtained at www.clinicaltrials.gov with the number of NCT05892094.

### Study population

Women were recruited from the outpatient clinics of the Department of Gynecology and Obstetrics at Koç University Hospital and Istanbul Medipol University, Istanbul, Türkiye, where the study was conducted. Participants were postmenopausal women aged 45–65 years who had experienced moderate or severe VMS, as determined by their responses to the vasomotor domain of the MENQOL questionnaire and the hot flushes/sweating item of the MRS. Women scoring ≥ 4 on any MENQOL vasomotor item (indicating moderate-to-severe bothersomeness) and/or ≥ 2 on the corresponding MRS item were classified as having moderate-to-severe VMS and were eligible for inclusion.

Additional inclusion criteria included engaging in ≤ 225 min of moderate-intensity physical activity per week, in accordance with the American College of Sports Medicine (ACSM) and World Health Organization (WHO) recommendations [15, 16], and having the physical ability to participate in resistance and aerobic training.

**Fig. 1:**
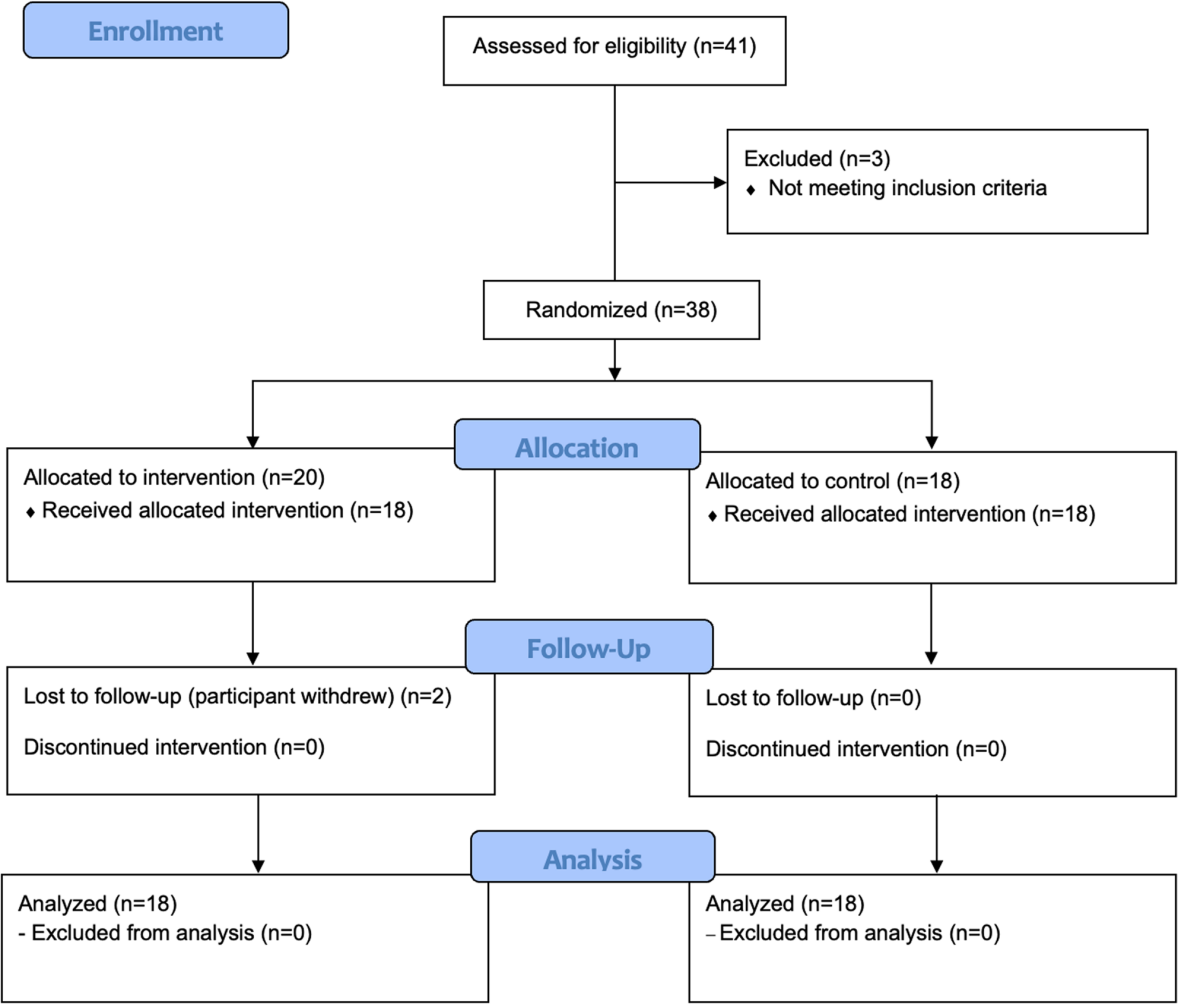
Flow of participants through the study

Participants who had used medical or hormone replacement therapy for the treatment of VMS within the past two months or who had uncontrolled hypertension (resting systolic blood pressure ≥ 160 mmHg and/or diastolic ≥ 100 mmHg) were excluded. Eligible women attended an outpatient clinic visit. During this visit, they received information about the study, provided written informed consent, and underwent eligibility screening and baseline clinical data collection. A total of 38 women were enrolled (Fig. 1).

Baseline clinical data, including age, body weight, height, and body mass index (BMI), were used to describe the characteristics of the participants and were not reassessed after the intervention. Randomization was performed at the second visit, after eligibility was confirmed based on VMS severity. Baseline questionnaires were completed at this visit. All measurements and questionnaires were repeated after 12 weeks. Prior to the final assessment, two participants in the exercise group withdrew for personal reasons. Recruitment took place between June 2023 and February 2024, spanning multiple seasons. According to local meteorological records, average temperatures during this period ranged from 10 °C to 28 °C, with relative humidity between 55% and 70%. This information was recorded to account for potential seasonal variation in VMS.

### Randomization and sample size

Randomization was performed using sealed and sequentially numbered envelopes. All procedures followed in accordance with the ethical standards of the responsible committee on human experimentation and with the Helsinki Declaration of 1975, as revised in 2008. The institutional review board at Istanbul Medipol University approved all recruitment and testing procedures (Reference no: E-10840098-772.02-754, decision no:97) on 26/01/2023. The study adhered to the CONSORT guidelines. Participants were fully informed about the purpose of the study and the experiments.

This study was designed to evaluate the effects of a combined resistance and aerobic exercise program on sleep quality, menopausal symptoms, and quality of life in postmenopausal women. Previous studies have reported moderate-to-large effects of exercise interventions on sleep and menopause-related outcomes. For instance, Qian et al. [17] showed that exercise interventions led to moderate-to-high improvements in sleep quality (standardized mean difference (SMD) = − 0.91, 95% confidence interval (CI): −1.45 to − 0.36), and Asghari et al. [18] found that aerobic exercise and nutrition education significantly reduced MENQOL and Greene scale scores. Based on these findings, the sample size for the present study was calculated using G*Power 3.1.9 (G*Power, Heinrich Heine University Düsseldorf, Germany). The following parameters were specified: a medium effect size (Cohen’s f = 0.25), a Type I error rate of α = 0.05, and a desired power of 1–β = 0.80. According to these parameters, a minimum of 17 participants per group was required. To account for potential dropouts, the study was designed to include 18 participants in each group, resulting in a final target sample size of 36.

### Intervention

**Fig. 2:**
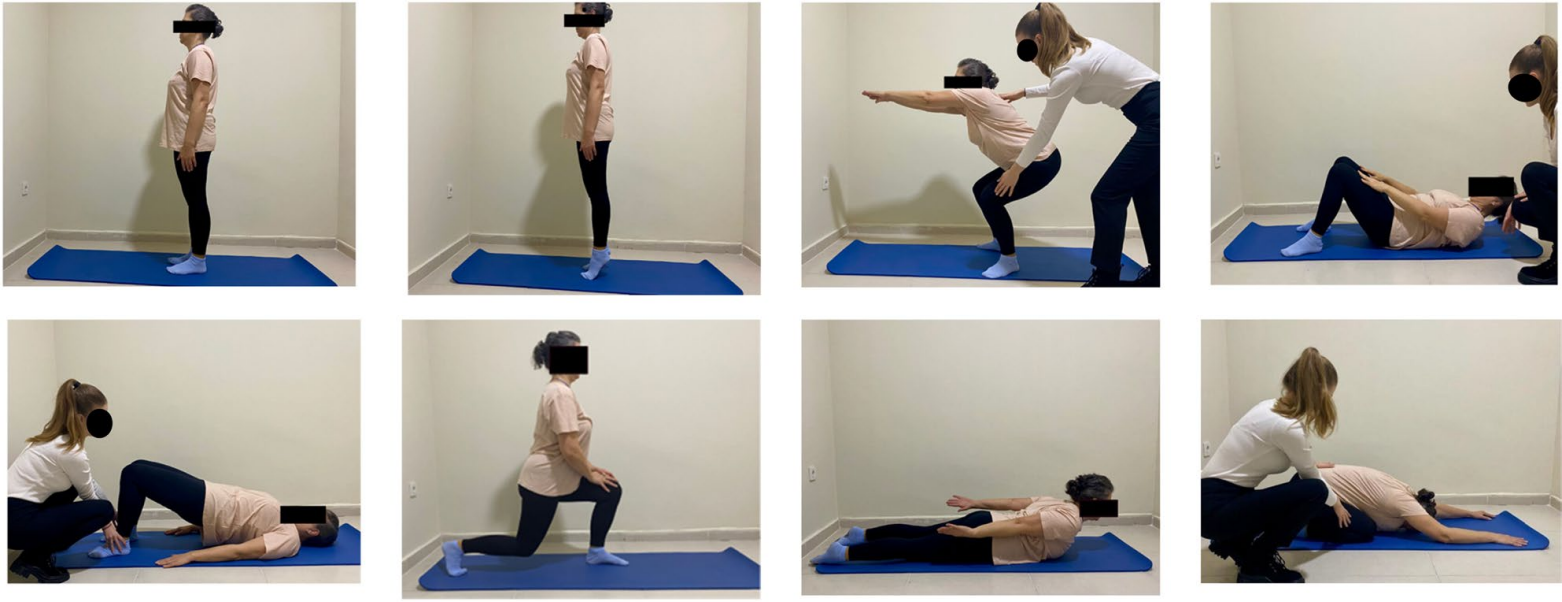
Resistance exercises

**Fig. 3:**
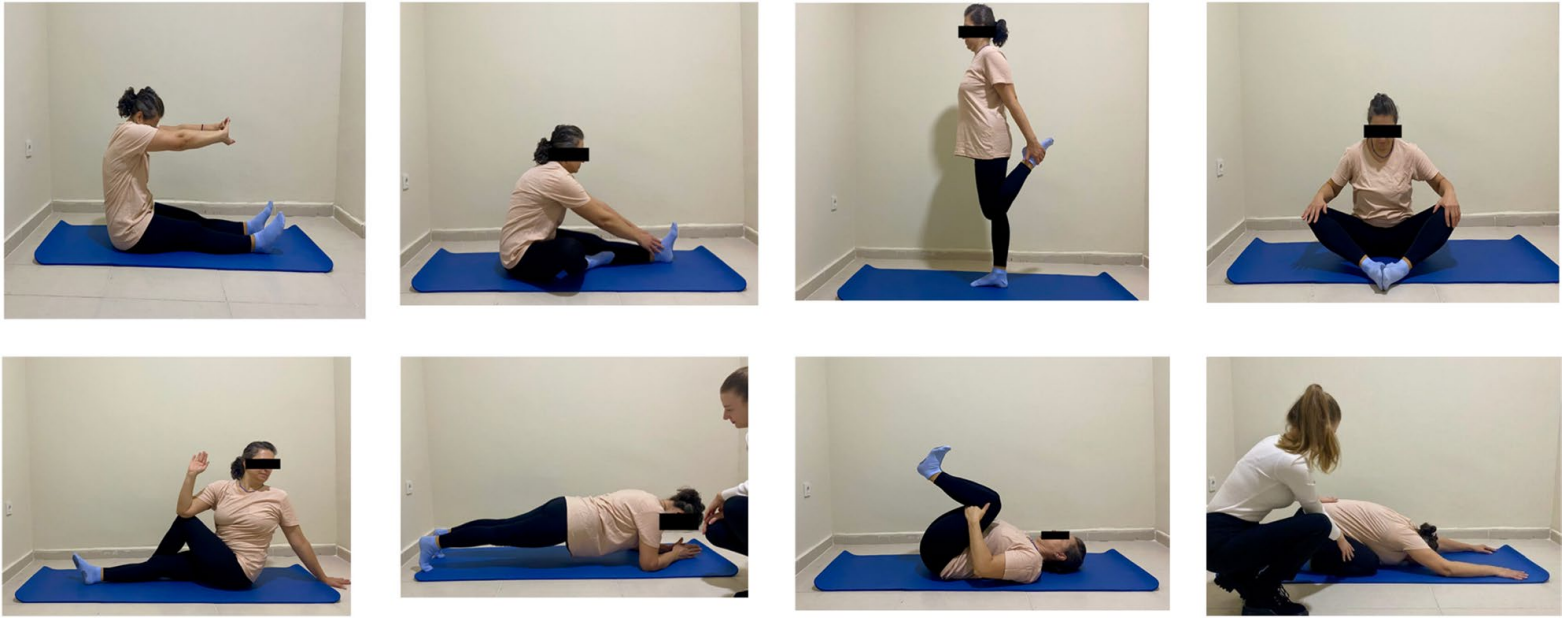
Stretching exercises

The 12-week resistance training program consisted of body-weight exercises, including push-ups, leg extensions, heel raises, squats, lunges, abdominal crunches, back raises and bridging exercises (Fig. 2). It was performed twice per week (24 sessions in total). Each session comprised a 5-minute warm-up, 40 min of resistance training and a 5-minute cool-down period including stretching exercises (Fig. 3). Progressive overload was implemented to ensure gradual adaptation and prevent training plateaus; training intensity and repetitions were adjusted according to the overload principle and monitored using the Borg Rating of Perceived Exertion (RPE) scale (0 = extremely easy; 20 = extremely hard). Participants were not permitted to exceed an RPE of 15, and 60 s of rest was provided between sets. Detailed descriptions of the exercises and progression can be found in the Supplementary Material.

In addition to the resistance training program, participants in the intervention group performed moderate-intensity aerobic walking three times per week on days when they did not have a resistance training session. The walking program was designed in accordance with ACSM guidelines [15]. Exercise intensity was determined using the validated talk test, whereby the ability to talk but not sing indicates a safe and sustainable moderate intensity [19, 20]. Perceived exertion was additionally monitored using the Borg RPE scale, and participants were instructed not to exceed an RPE of 15. The program was structured to increase progressively: walking duration was 30 min per session during weeks 1–3, 35 min during weeks 4–7, and 40 min during weeks 8–12. Each session included a 10-minute warm-up and cool-down at a comfortable pace [21]. To ensure adherence to the program, safety and proper technique, both resistance and aerobic sessions were supervised by the same physiotherapist. Attendance and completion of each session were recorded for every participant. Safety precautions, such as the Borg RPE and the Talk Test, were applied throughout the supervised exercise sessions to ensure the appropriate intensity level and the safety of the participants. Participants in both the exercise and control groups were instructed to maintain their usual level of physical activity and to refrain from starting any new exercise or treatment for VMS during the 12-week study. Adherence to these instructions was verified via biweekly telephone calls.

### Outcome assessments

The primary outcome was the change in VMS severity from baseline to week 12, as assessed using the vasomotor domain of the MENQOL questionnaire. As the study relied on validated self-reported questionnaires rather than daily symptom diaries, the frequency of VMS episodes was not recorded; therefore, severity scores were used as the primary indicator of VMS burden. Secondary outcomes included changes in quality of life and sleep quality, which were assessed using the MENQOL, MRS, and PSQI.

The MENQOL is a 29-item questionnaire originally developed by Lewis et al. [22] to assess the severity and presence of menopausal symptoms as well as quality of life. Each item is answered on a 6-point scale, and scores are generated in four domains of menopausal symptoms, as experienced over the last month: vasomotor, psychosocial, physical, and sexual. As the score increases, the severity of the complaint also increases. The vasomotor domain was used as the primary outcome to evaluate VMS. This domain consists of three items assessing hot flushes, night sweats, and sweating experienced over the past month. Participants first indicate whether they experienced each symptom (“yes” or “no”), and if present, rate how bothersome the symptom has been on a 7-point scale (0–6). A score of 0 indicates that the symptom was not experienced; 1 indicates that it was present but not bothersome, and 2–6 represent increasing degrees of bother or severity. Scores from the three items are averaged to obtain the vasomotor domain score, which reflects symptom severity rather than frequency. The Turkish version has shown good reliability and has been validated for the assessment of MENQOL in postmenopausal women [23].

The MRS was originally developed by Heinemann et al. as a validated questionnaire consisting of 11 items grouped into three categories – somatic, psychological, and urogenital [24]. The Turkish version of the MRS was used to assess the severity of menopausal complaints and their impact on women’s quality of life [25]. Each item is a menopausal symptom graded on a 5-point Likert scale with a minimum score of 0 and a maximum of 4. Each symptom is rated from 0 to 4 as ‘no symptom’, ‘mild’, ‘moderate’, and ‘severe’ respectively.

Sleep quality was assessed with the PSQI, which was originally developed by Buysse et al. to measure sleep quality and disturbances over a 1-month time interval [26]. The Turkish version has demonstrated good validity and reliability [27]. The 19 items in the index are categorized into seven components, each evaluating different aspects of sleep. The PSQI comprises seven components assessing various aspects of sleep. Each item is scored from 0 (no difficulty) to 3 (severe difficulty), yielding a total score between 0 and 21. A score of ≤ 5 indicates good sleep quality.

### Statistical analyses

All statistical analyses were performed using IBM SPSS Statistics version 26 (IBM Corp. Armonk, NY, USA). Descriptive statistics (mean ± standard deviation, frequency, and percentage) were used to summarize baseline characteristics. To evaluate the effects of the intervention over time, a two-way mixed repeated-measures ANOVA was conducted for each outcome, with group (intervention vs. control) as the between-subjects factor and time (pre-test vs. post-test) as the within-subjects factor.

When significant main effects or group × time interactions were detected, Bonferroni-corrected pairwise comparisons were performed to identify the source of differences. Effect sizes were calculated using partial eta squared (η²) and interpreted according to conventional thresholds.

All assumptions of mixed ANOVA, including normality, homogeneity of variance, and sphericity, were assessed prior to analysis. The results were presented in tables of descriptive statistics and ANOVA outputs, and the time-related changes by the group were illustrated using line graphs. Statistical significance was set at *p* < 0.05.

## Results

In total, 38 women were randomized, and 36 women completed the 12-week study period (Fig. 1). Two participants in the exercise group did not fill out the questionnaires at week 12. The mean age of the women who participated in the study was 52 years, the mean age at menopause was 49 years and the mean body mass index was 25 kg/m2. There were no differences between groups in any of the baseline variables (Table 1).

**Table 1:** Baseline data of included participants

	Intervention group (*n* = 18)		Control Group (*n* = 18)		*p*-value
	Mean ± SD	min-max	Mean ± SD	min-max	
Age at inclusion, years	51.81 ± 4.67	44.00–60.00	52.50 ± 4.42	44.00–62.00	0.63
Weight, kg	66.76 ± 6.23	56.00–77.00	66.94 ± 5.84	54.00–75.00	0.91
Body mass index, kg/m2	24.83 ± 2.42	21.45–32.05	25.22 ± 2.24	22.15–28.93	0.54
Number of pregnancies	2 (1–3)	0–7	2 (1–3)	1–4	0.89
Number of births	1 (1–2)	0–3	1 (1–2)	1–3	0.72
Number of abortions	0 (0–1)	0–5	1 (0–1)	0–2	0.31
Age of menopause	48.25 ± 4.15	44–53	49.05 ± 2.83	44–54	0.42

*SD* Standard Deviation, *p*-values represent between-group comparisons using Welch’s *t*-test (continuous variables) or Mann–Whitney U test (count variables)

### Changes in MENQOL vasomotor domain

For the vasomotor domain of the MENQOL scale, the mixed repeated-measures ANOVA revealed a significant main effect of group (*F*(1,11.64) = 7.41, *p* < 0.001, η²=0.188). This indicates that the intervention and control groups performed significantly differently overall. Examination of the mean scores showed that the intervention group (M ± SD = 4.83 ± 1.07) had higher scores compared to the control group (M ± SD = 4.50 ± 0.94) (Table 2).

**Table 2:** Descriptive statistics and mixed repeated-measures ANOVA results for MENQOL vasomotor domain across time and groups

Test	Group	Mean ± SD	Effects	F (df1, df2)	*p*-value	η²	Significant Differences (Bonferroni)
Pre-test^1^	Control^a^	4.50 ± 0.94	Group	F(1;11,64) = 7.41	0.0001	0,188	a > b
	Intervention^b^	4.83 ± 1.07	Time	F(1;14,39) = 87.71	0.0001	0,733	1 > 2
Post-test^2^	Control^a^	4.74 ± 0.92	Group × Time	F(1;22,90) = 139.6	0.0001	0,814	Intervention:1 > 2
	Intervention^b^	2.75 ± 0.77					

*SD* Standard Deviation, *F* Mix ANOVA, *η² *partial eta squared (effect size)

**Fig. 4:**
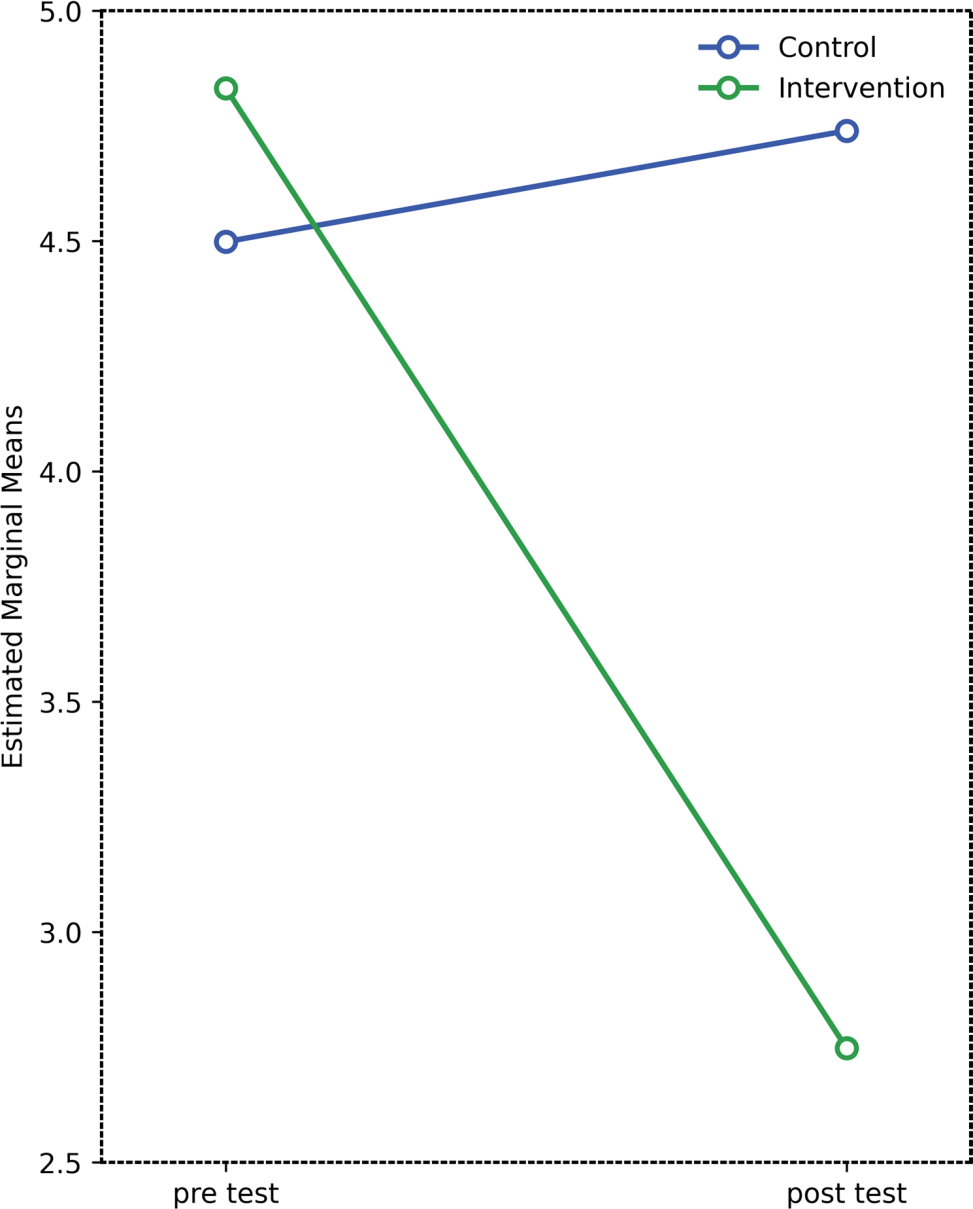
Change in MENQOL vasomotor scores over time in intervention and control groups

A significant main effect of time was also found (F(1,14.39) = 87.71, *p* < 0.001, η²=0.733), indicating that participants’ scores changed significantly across the two measurements (pre-test vs. post-test). Bonferroni pairwise comparisons revealed that pre-test scores were significantly higher than post-test scores (1 > 2) (Fig. 4).

Furthermore, the group × time interaction was statistically significant (F(1,22.90) = 139.6, *p* < 0.001, η²=0.814), indicating that the two groups exhibited different patterns of change over time. Post hoc Bonferroni tests revealed that pre-test scores were significantly higher than post-test scores in the intervention group (Intervention: 1 > 2) (Table 2; Fig. 4).

Overall, these results suggest that participants in the intervention group experienced a significant improvement over time, whereas no comparable change was observed in the control group. This finding supports the effectiveness of the intervention program implemented.

### Changes in MENQOL other domains (psychosocial, physical, sexual)

In addition to the vasomotor findings, the results of the mixed repeated-measures ANOVA for the other MENQOL domains showed similar patterns with varying levels of significance. For the psychosocial domain, a significant main effects of group (*F*(1,21.97) = 6.22, *p* < 0.001, η²=0.163) and time (*F*(1,6.41) = 11.13, *p* < 0.001, η²=0.258) were observed, as well as a significant group × time interaction (*F*(1,12.20) = 21.18, *p* < 0.001, η²=0.398) (Table 3). Corresponding temporal changes are provided in Supplementary Figure S1.

**Table 3:** Descriptive statistics and mixed repeated-measures ANOVA results for MENQOL psychosocial domain across time and groups

Test	Group	Mean ± SD	Effects	F (df1, df2)	*p*-value	η²	Significant Differences (Bonferroni)
Pre-test^1^	Control^a^	3,39 ± 1,37	Group	F(1;21,97)=,6,22	0.0001	0,163	a > b
	Intervention^b^	3,10 ± 1,88	Time	F(1;6,41) = 11,13	0.0001	0,258	1 > 2
Post-test^2^	Control^a^	3,63 ± 1,40	Group × Time	F(1;12,20) = 21,18	0.0001	0,398	Intervention:1 > 2
	Intervention^b^	1,64 ± 0,95					

*SD* Standard Deviation, *F* Mix ANOVA, *η²* partial eta squared (effect size)

For the physical domain, the main effect of group was not significant (*F* (1,32) = 8.49, *p* > 0.05), while the effect of time was significant (*F* (1,32) = 10.41, *p* < 0.001, η²=0.245). A significant group × time interaction was also found (*F* (1,32) = 29.14, *p* < 0.001, η²=0.477) (Table 4). The trajectory of physical scores is shown in Supplementary Figure S2.

**Table 4:** Descriptive statistics and mixed repeated-measures ANOVA results for MENQOL physical domain across time and groups

Test	Group	Mean ± SD	Effects	F (df1, df2)	*p*-value	η²	Significant Differences (Bonferroni)
Pre-test^1^	Control^a^	3,04 ± 1,28	Group	F(1;32) = 8,49	0,142		
	Intervention^b^	3,07 ± 1,83	Time	F(1;32) = 10,41	0.0001	0,245	1 > 2
Post-test^2^	Control^a^	3,34 ± 1,22	Group × Time	F(1;32) = 29,14	0.0001	0,477	Intervention:1 > 2
	Intervention^b^	1,90 ± 1,32					

*SD* Standard Deviation, *F* Mix ANOVA, *η² *partial eta squared (effect size)

Regarding the sexual domain, neither the main effects of group (*F* (1,32) = 0.49, *p* > 0.05) nor time (*F* (1,32) = 0.66, *p* > 0.05) were significant. However, a significant group × time interaction was observed (*F* (1,32) = 6.01, *p* < 0.05, η²=0.158), suggesting that the intervention group experienced a modest but significant reduction in scores over time (Intervention:1 > 2) (Table 5). The temporal profile for this domain is presented in Supplementary Figure S3.

**Table 5:** Descriptive statistics and mixed repeated-measures ANOVA results for MENQOL sexual domain across time and groups

Test	Group	Mean ± SD	Effects	F (df1, df2)	*p*-value	η²	Significant Differences (Bonferroni)
Pre-test^1^	Control^a^	2,41 ± 1,50	Group	F(1;32) = 0,49	0,48		
	Intervention^b^	3,25 ± 2,57	Time	F(1;32) = 0,66	0,42		
Post-test^2^	Control^a^	2,67 ± 1,41	Group × Time	F(1;32) = 6,01	0,02	0,158	Intervention:1 > 2
	Intervention^b^	2,73 ± 2,12					

*SD* Standard Deviation, *F* Mix ANOVA, *η² *partial eta squared (effect size)

Overall, these findings suggest that the intervention program produced meaningful improvements in the psychosocial and physical aspects of menopausal quality of life, with less pronounced but still favourable changes observed in the sexual domain.

### Changes in MRS somatic domain

**Fig. 5:**
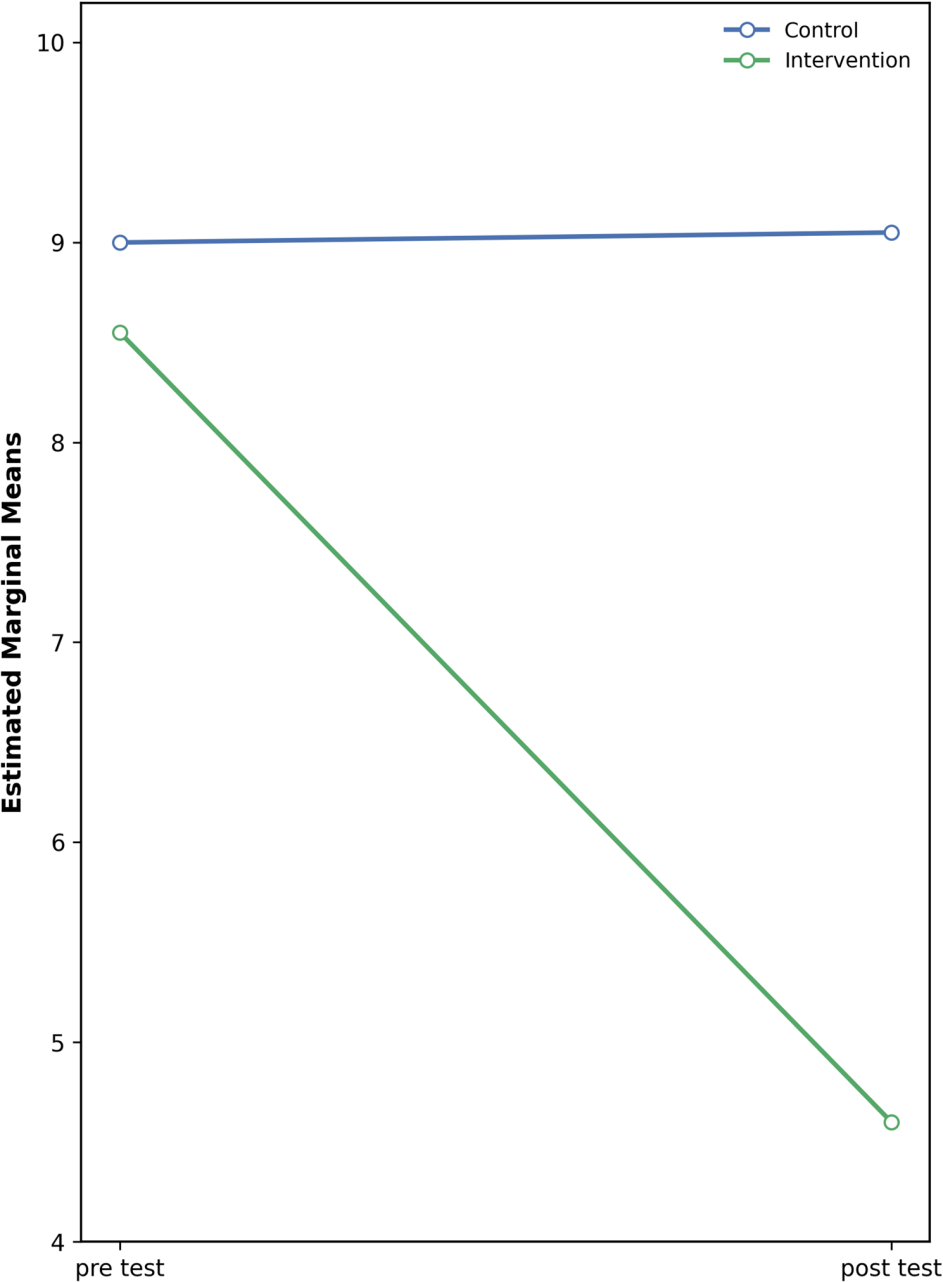
Change in MRS somatic scores over time in intervention and control groups

For the somatic domain of the MRS scale, the mixed repeated-measures ANOVA revealed that the main effect of group was not statistically significant (F (1,32) = 2.93, *p* > 0.05), indicating that the intervention and control groups demonstrated similar overall symptom levels. However, mean scores showed slightly lower somatic symptom levels in the intervention group (M ± SD = 8.56 ± 4.86) than in the control group (M ± SD = 9.00 ± 3.93), though this difference was not statistically significant (Table 6). In contrast, a significant main effect of time was found (F (1,32) = 63.82, *p* < 0.001, η² = 0.603), indicating that participants’ symptom scores changed substantially from pre-test to post-test. Bonferroni post hoc analysis showed that pre-test scores were significantly higher than post-test scores (1 > 2). The temporal change in somatic symptoms is illustrated in Fig. 5.

**Table 6:** Descriptive statistics and mixed repeated-measures ANOVA results for MRS somatic domain across time and groups

Test	Group	Mean ± SD	Effects	F (df1, df2)	*p*-value	η²	Significant Differences (Bonferroni)
Pre-test^1^	Control^a^	9,00 ± 3,93	Group	F(1;32) = 2,93	0,09		
	Intervention^b^	8,56 ± 4,86	Time	F(1;32) = 63,82	0.0001	0,603	1 > 2
Post-test^2^	Control^a^	9,06 ± 4,40	Group × Time	F(1;32) = 67,53	0.001	0,617	Intervention:1 > 2
	Intervention^b^	4,63 ± 3,61					

*SD* Standard Deviation, *F* Mix ANOVA, *η² *partial eta squared (effect size)

Additionally, the group × time interaction was statistically significant (F (1,32) = 67.53, *p* < 0.01, η²=0.617), demonstrating that the pattern of somatic symptom change over time differed between groups. Specifically, post hoc comparisons showed that the intervention group exhibited a significant decrease in somatic symptoms from pre-test to post-test (intervention group: 1 > 2) (Table 6; Fig. 5).

### Changes in MRS psychological and urogenital domains

For the psychological domain of the MRS scale, the mixed repeated-measures ANOVA revealed that the main effect of group was not significant (F (1,32) = 2.71, *p* > 0.05), indicating comparable psychological symptom levels between the intervention and control groups. However, mean scores were slightly higher in the intervention group (M ± SD = 9.88 ± 3.70) than in the control group (M ± SD = 8.56 ± 2.33), though this difference was not statistically significant (Table 7).

**Table 7:** Descriptive statistics and mixed repeated-measures ANOVA results for MRS psychological domain across time and groups

Test	Group	Mean ± SD	Effects	F (df1, df2)	*p*-value	η²	Significant Differences (Bonferroni)
Pre-test^1^	Control^a^	8,56 ± 2,33	Group	F(1;32) = 2,71	0,109		1 > 2
	Intervention^b^	9,88 ± 3,70	Time	F(1;32) = 36,97	0.0001	0,536	Control:1 < 2
Post-test^2^	Control^a^	9,72 ± 2,47	Group × Time	F(1;32) = 108,53	0,02	0,772	Intervention:1 > 2
	Intervention^b^	5,44 ± 2,25					

*SD* Standard Deviation, *F* Mix ANOVA, *η² *partial eta squared (effect size)

A significant main effect of time was observed (F (1,32) = 36.97, *p* < 0.001, η²=0.536), indicating an overall reduction in psychological symptoms from pre- to post-test. Notably, the group × time interaction was also significant (F (1,32) = 108.53, *p* < 0.05, η²=0.772), indicating that the two groups changed in opposite directions over time. Post hoc analyses revealed that, while psychological scores decreased in the intervention group (1 > 2), they increased in the control group (control group: 1 < 2). The temporal patterns are presented in Supplementary Figure S4.

For the urogenital domain, neither the group effect (F (1,32) = 2.93, *p* > 0.05) nor the time effect (F (1,32) = 0.48, *p* > 0.05) was significant, indicating no meaningful overall differences between groups or across time. The intervention group showed slightly higher mean scores (M ± SD = 5.00 ± 3.71) than the control group (M ± SD = 4.11 ± 3.22), but these differences were not statistically significant (Table 8).

**Table 8:** Descriptive statistics and mixed repeated-measures ANOVA results for MRS urogenital domain across time and groups

Test	Group	Mean ± SD	Effects	F (df1, df2)	*p*-value	η²	Significant Differences (Bonferroni)
Pre-test^1^	Control^a^	4,11 ± 3,22	Group	F(1;32) = 2,93	0,09		
	Intervention^b^	5,00 ± 3,71	Time	F(1;32) = 0,48	0,49		
Post-test^2^	Control^a^	5,06 ± 2,96	Group × Time	F(1;32) = 11,77	0,001	0,269	Control:1 < 2
	Intervention^b^	4,38 ± 3,46					

*SD* Standard Deviation, *F* Mix ANOVA, *η²* partial eta squared (effect size)

However, the group × time interaction was significant (F (1,32) = 11.77, *p* < 0.01, η²=0.269), suggesting different symptom trajectories for the two groups. Post hoc comparisons revealed that psychological symptoms increased from pre- to post-test in the control group (Control:1 < 2), whereas the intervention group did not exhibit a comparable change. The temporal change is displayed in Supplementary Figure S5.

### Changes in PSQI

For the PSQI total score, the mixed repeated-measures ANOVA indicated that the main effect of group was not statistically significant (F (1,32) = 1.14, *p* > 0.05), suggesting similar overall sleep quality between the intervention and control groups. Mean scores were slightly higher in the intervention group (M ± SD = 8.25 ± 4.73) compared to the control group (M ± SD = 8.06 ± 4.36), but this difference was not meaningful (Table 9).

**Table 9:** Descriptive statistics and mixed repeated-measures ANOVA results for PSQI domain across time and groups

Test	Group	Mean ± SD	Effects	F (df1, df2)	*p*-value	η²	Significant Differences (Bonferroni)
Pre-test^1^	Control^a^	8,06 ± 4,36	Group	F(1;32) = 1,14	0,29		
	Intervention^b^	8,25 ± 4,73	Time	F(1;32) = 14,63	0.0001	0,314	1 > 2
Post-test^2^	Control^a^	8,44 ± 4,15	Group × Time	F(1;32) = 24,65	0.0001	0,435	Interventon:1 > 2
	Intervention^b^	5,25 ± 3,42					

*SD* Standard Deviation, *F* Mix ANOVA, *η²* partial eta squared (effect size)

**Fig. 6:**
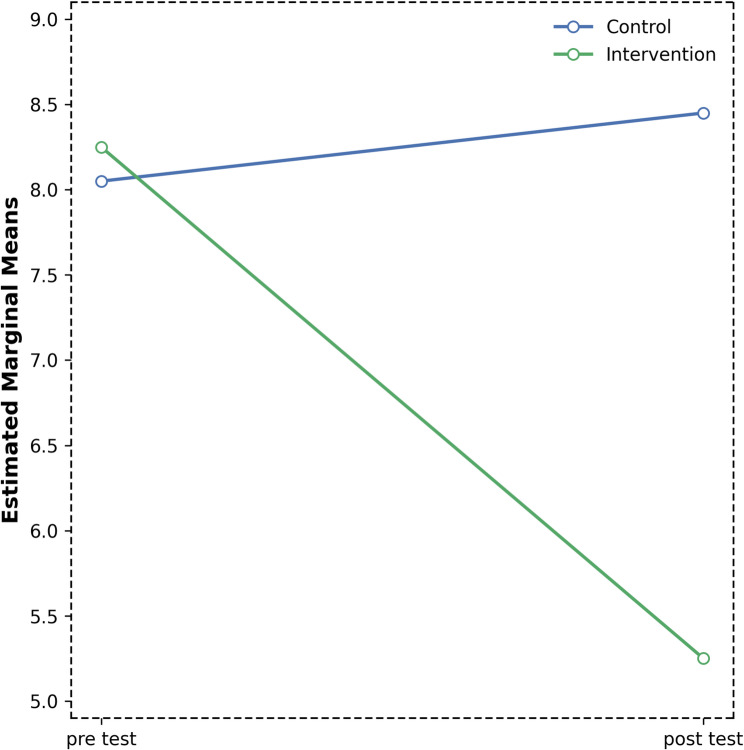
Change in PSQI scores over time in intervention and control groups

A significant main effect of time was found (F (1,32) = 14.63, *p* < 0.01, η²=0.314), indicating an overall improvement in sleep quality from pre- to post-test, with pre-test scores significantly higher than post-test scores (1 > 2). The temporal change in PSQI scores is illustrated in Fig. 6.

The group × time interaction was also significant (F (1,32) = 24.65, *p* < 0.01, η²=0.435), demonstrating differing trajectories between the two groups. Post hoc comparisons showed that the intervention group experienced a significant reduction in PSQI scores from pre- to post-test (Intervention:1 > 2), whereas the control group did not exhibit a comparable improvement (Table 9; Fig. 6).

## Discussion

This study evaluated the effects of a 12-week resistance and aerobic exercise program on menopausal symptoms, particularly the severity of VMS. The intervention produced clinically meaningful reductions in VMS severity and improvements in sleep quality and psychosocial and physical domains. These findings suggest that structured exercise is a safe and feasible non-pharmacological option for postmenopausal women. Although hormone therapy is the most effective treatment for VMS, its use is limited by contraindications and patient preference, increasing interest in alternative approaches such as exercise. Previous studies have reported mixed effects of exercise on VMS severity, often attributed to differences in program intensity, duration and adherence [7]. Our results contribute to the growing evidence suggesting that well-supervised, progressively structured programmes can produce meaningful improvements.

The observed improvements are consistent with those reported in earlier trials, which found that exercise participation enhanced quality of life, physical functioning and emotional well-being [28–31]. Bodyweight resistance training is widely used in rehabilitation and is considered safe, accessible and appropriate for middle-aged and older adults [32]. Furthermore, observational studies show that physically active women experience less severe VMS than sedentary individuals, which reinforces the importance of promoting regular physical activity. Progressive, low-intensity exercise is known to improve strength, functional capacity, and bone health while posing minimal risk [31]. Nevertheless, inconsistent findings across intervention studies emphasise the impact of exercise intensity, compliance, and dropout rates on outcomes [33].

This pattern of improvement is consistent with the findings of Dabrowska-Galas et al. [34], who reported an inverse relationship between higher physical activity levels and hot flushes and sleep disturbances. Previous intervention trials, including resistance training leading to a 44% reduction in moderate-to-severe hot flushes, and combined aerobic programmes producing a 50% improvement on average, suggest that intensity and consistency may be key factors in reducing symptoms. In our study, beginning with low loads and adjusting repetitions according to participant tolerance may have encouraged adherence and contributed to the improvements observed.

Exercise has also been shown to enhance the psychological and physical aspects of quality of life [35]. Several studies, including those by Elavsky et al. [14] and Duijts et al. [36], have demonstrated improvements in symptoms and quality of life following aerobic, walking-based and supervised home programmes. Evidence from various modalities, such as yoga and structured physical activity, highlights the importance of well-designed interventions for this population.

It has been documented that early postmenopausal women experience broader benefits across the musculoskeletal, cardiorespiratory and metabolic fitness domains [37, 38]. Asikainen et al. [39] found that combining moderate-intensity walking with resistance training produced a wide range of functional improvements. The present findings, which indicate improvements across MENQOL domains, support these observations. Several mechanisms have been proposed to explain exercise-related reductions in VMS, including enhanced autonomic balance, increased parasympathetic activity, improved beta-endorphin release and more stable thermoregulation. Neurophysiological evidence implicating KNDy neurons in thermoregulatory disruption provides a potential biological explanation for these effects [40]. Although these mechanisms may contribute to the observed effects, they were not assessed directly in this study and should therefore be interpreted with caution.

Combining walking with resistance exercise has also been shown to improve sleep quality, physical functioning, and psychological well-being [41]. As many women prefer behavioural approaches to medication, addressing misconceptions such as the belief that exercise worsens VMS symptoms may encourage participation. Although the effects on VMS remain inconsistent, therapeutic walking programmes have demonstrated improvements in menopause-related outcomes [42]. Additional support comes from resistance-based programmes that improve muscle quality and functional fitness. These programmes align with the present results [43]. Qualitative findings describing reductions in VMS severity, enhanced well-being, and sustained increases in physical activity following resistance training further reinforce its potential benefits [44, 45].

The minimal clinically important difference of one point was exceeded in all domains except the sexual domain. This is to be expected, given the strong link between sexual and urogenital symptoms, and oestrogen deficiency [46, 47]. Despite the well-documented benefits of exercise, many women find it challenging to sustain long-term physical activity, highlighting the need for structured and supervised programmes to improve adherence. Although the study period spanned multiple seasons, continuous recruitment and assessment probably reduced the impact of seasonal variation on VMS severity.

Given their safety, accessibility, and low resource requirements, the exercise components used here could be integrated into physiotherapy and community health practices as a practical symptom management option. Future research should examine long-term outcomes through extended interventions or follow-up assessments, compare different training modalities and delivery formats, and evaluate whether combining exercise with menopausal hormone therapy provides any additional benefits. Incorporating objective measures of VMS frequency and relevant hormonal or physiological markers would also help to clarify the underlying mechanisms.

### Limitations of the study

This study has several limitations. VMS were assessed subjectively, and no hormonal or physiological markers were collected. The sample size was modest, and the lack of long-term follow-up limits conclusions about the durability of the effects. Generalizability is restricted because participants were women aged 45–65 recruited from university outpatient clinics. Although adherence and exercise intensity were not monitored objectively, all sessions were supervised using validated non-invasive tools. Additionally, BMI and body weight were recorded only at baseline; future studies should include post-intervention anthropometric and physiological assessments to clarify underlying mechanisms.

## Conclusion

A 12-week combined resistance and aerobic exercise program significantly reduced VMS severity and improved overall well-being in postmenopausal women, supporting structured exercise as a safe and practical non-pharmacological intervention.

## Supplementary Information

Supplementary Material 1.

Supplementary Material 2.

## Data Availability

The datasets generated during and/or analyzed during the current study are available from the corresponding author on reasonable request.
