# Supplementary material for: Effects of exercise training on vasomotor symptoms and quality of life in postmenopausal women: a randomized controlled trial
Source: BMC Womens Health. 2025 Dec 22;25:612. doi: 10.1186/s12905-025-04231-y (PMC12750805; doi:10.1186/s12905-025-04231-y)
Supplement: Supplementary file 1 — Supplementary Material 1. [file 12905_2025_4231_MOESM1_ESM.docx]

**Supplementary material 1.** Resistance exercise program

| **Exercise program** | **Type and duration** | **Region** | **Contents** |
| --- | --- | --- | --- |
| Warm-up | Stretching (5 min) | Whole body | Stretching, walking |
| Main exercise | Resistance  (30 min) | Upper body | Crunch, Wall push-ups, Back extension, Plank, Shoulder flexion-extension, Elbow flexion-extension |
|  |  | Lower body | Squat, Lunge, Leg abduction, Bridge, Toe-heel raises |
| Cool-down | Stretching (5 min) | Whole body | Stretching |

**Supplementary material 2.** Resistance exercise protocol and progression

| Weeks | Number of repetitions | Sets | Intensity (Borg Scale) | Resistance exercises |
| --- | --- | --- | --- | --- |
| 1 | 6 | 3 | 9 | Squat, wall push-ups, toe-heel raises, crunch, bridge, shoulder flexion, elbow flexion-extension |
| 2 | 8 | 3 | 10 | Squat, wall push-ups, back extension, crunch, bridge, shoulder flexion, elbow flexion-extension, toe-heel raises |
| 3 | 10 | 3 | 11 | Squat, back extension, crunch, bridge, shoulder flexion, elbow flexion-extension, toe-heel raises |
| 4 | 12 | 3 | 12 | Back extension, crunch, bridge, shoulder flexion, elbow flexion-extension, toe-heel raises |
| 5 | 10 | 4 | 13 | Squat, crunch, lunge, single leg bridge, shoulder flexion, elbow flexion-extension, toe-heel raises |
| 6 | 10 | 4 | 13 | Squat, crunch, lunge, single leg bridge, shoulder flexion, elbow flexion-extension |
| 7 | 12 | 5 | 14 | Squat, crunch, opposite arm and leg raise, half push-up, lunge, single leg bridge, shoulder flexion, elbow flexion-extension |
| 8 | 12 | 5 | 14 | Squat, crunch, opposite arm and leg raise, half push-up, lunge, single leg bridge, shoulder flexion, elbow flexion-extension |
| 9 | 15 | 3 | 15 | Squat, crunch, opposite arm and leg raise, half push-up, lunge, single leg bridge, shoulder flexion, elbow flexion-extension |
| 10 | 15 | 3 | 15 | Squat, crunch, opposite arm and leg raise, half push-up, lunge, single leg bridge, shoulder flexion, elbow flexion-extension |
| 11 | 15 | 3 | 15 | Squat, crunch, opposite arm and leg raise, half push-up, lunge, single leg bridge, shoulder flexion, elbow flexion-extension, plank |
| 12 | 15 | 3 | 15 | Squat, crunch, opposite arm and leg raise, half push-up, lunge, single leg bridge, shoulder flexion, elbow flexion-extension, plank |
