# Supplementary material for: Effects of exercise training on vasomotor symptoms and quality of life in postmenopausal women: a randomized controlled trial
Source: BMC Womens Health. 2025 Dec 22;25:612. doi: 10.1186/s12905-025-04231-y (PMC12750805; doi:10.1186/s12905-025-04231-y)
Supplement: Supplementary file 2 — Supplementary Material 2. [file 12905_2025_4231_MOESM2_ESM.docx]

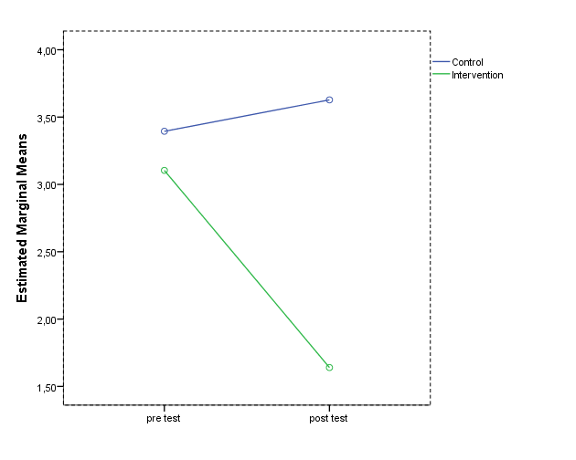


**Figure S1.** Change in MENQOL psychosocial scores over time in intervention and control groups.


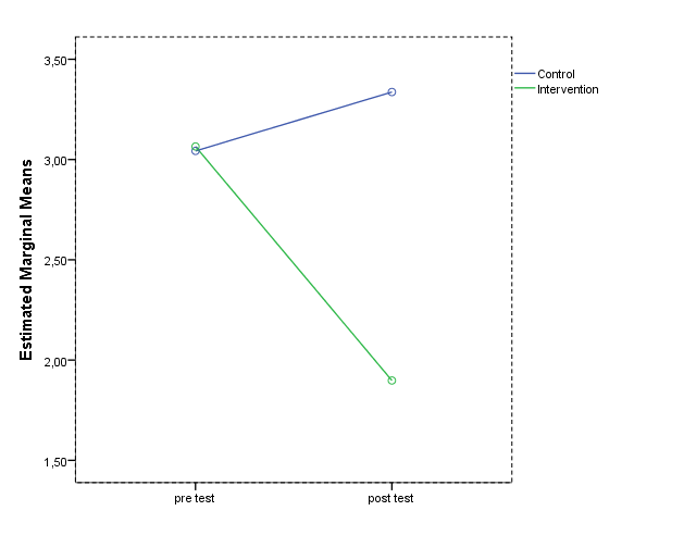


**Figure S2.** Change in MENQOL physical scores over time in intervention and control groups.


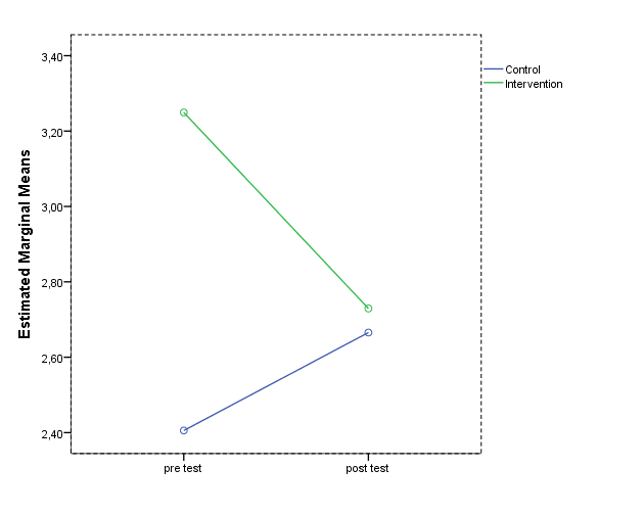


**Figure S3.** Change in MENQOL sexual scores over time in intervention and control groups.


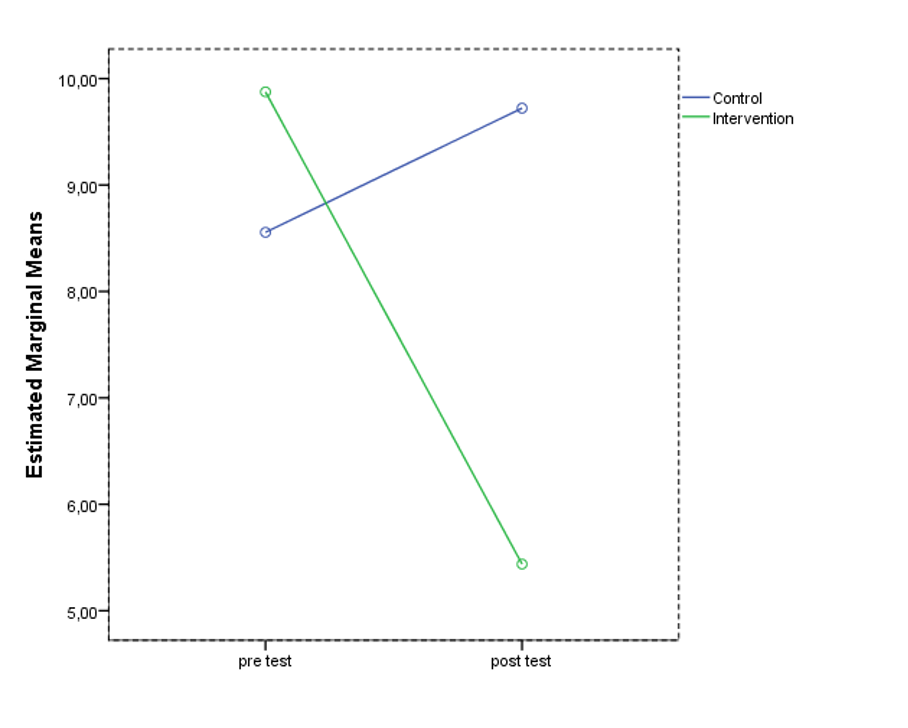


**Figure S4.** Change in MRS psychological scores over time in intervention and control groups.


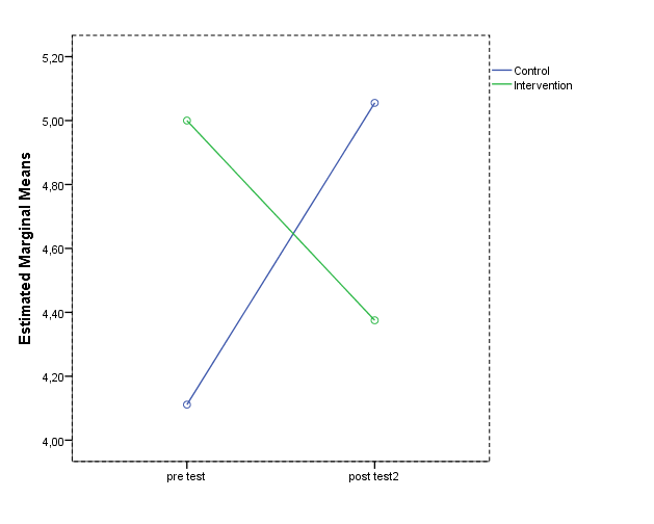


**Figure S5.** Change in MRS urogenital scores over time in intervention and control groups.
